# Histology, imaging and new diagnostic work-flows in pathology

**DOI:** 10.1186/1746-1596-3-S1-S14

**Published:** 2008-07-15

**Authors:** John Gilbertson, Yukako Yagi

**Affiliations:** 1 The Pathology Service, Massachusetts General Hospital, Boston, MA, USA

## Abstract

**Introduction:**

Since their introduction in 1999, fully automated, high speed, high-resolution whole slide imaging devices have become increasing more reliable, fast and capable. While by no means perfect, these devices have evolved to a point where one can consider placing them in a pre-diagnostic role in a clinical histology lab.

**Methods:**

At the Massachusetts General Hospital, we are running a pilot study placing high end WSI devices in our main clinical histology lab (after the cover slipper and before slides are sent to the pathologist) to examine the requirement for both the machine and the laboratory.

**Results:**

Placing WSI systems in the clinical lab stresses the system in terms of reliability and throughput. Significantly however, success requires significant modification to the lab workflow. It is likely laboratories need to move from manual, large batch processes to increasingly automated, continuous flow (or mini-batch) processes orchestrated by the LIS using bar coding to track and direct slides, and incorporating the decision to image into the specimen type and the histology orders. Furthermore, image quality, capture speed and reliability are functions of the quality of the histology presented to the WSI devices.

**Conclusion:**

Imaging in pathology does not begin in a WSI robot but in the grossing room and in the histology lab. As more and more imaging devices are placed in histology lab, the inter-relationships histology and pathology imaging will become increasing understood.

## Introduction

Fully automated, high speed, high-resolution whole slide imaging (WSI) systems have been available for about 8 years [1]. The early development of these systems focused on the basic technical issues surrounding the creation of high quality images at reasonable speed and the management and display and the very large data sets they created. While these issues have by no means been completely solved, multiple companies around the world are now producing effective systems based on traditional microscope optics as well as more radical designs [2]; investigators have demonstrated that, in some situations, pathologists examining images rapidly captured by fully automated WSI robots can make diagnoses that are as correct and as complete as those made directly from the glass [3]. However, the WSI experience is not yet as good as that of the microscope (diagnostic capability is a poor proxy for image quality) and serious problems with WSI diagnosis have been reported in some specimen types [4]. That said, the progress in whole slide image capture has been remarkable given the technical issues involved, and we expect further improvement in the future, both through general, relentless growth of technology driven by Moore's Law and new microscope optics, stage and camera designs specifically for WSI.

While the technical issues surrounding WSI are challenging and fascinating, to be successful, WSI must be a useful tool to enhance pathologic examination of tissue. If we can successfully digitize our slides, we can apply computational power and network connectivity, the drivers of productivity in modern world, to the analysis of tissue morphology and the practice of anatomic pathology. With reliable – if not perfect – systems available, we can now turn to the question of how large scale WSI can be implemented in Pathology practices.

This paper is about our initial attempt of implementing WSI in the clinical histology laboratory – and the somewhat surprising things we discovered.

## Methods

Today, the majority of WSI work (at MGH and elsewhere) is "post-diagnostic" in that they occur after the pathologist has used the glass slide to make the diagnosis. They include conference support, teaching, research (especially support of morphologic, immunohistochemistry molecular analysis and tissue banking). Other institutions have reported at least pilot studies in surgical pathology QA [5]. These applications make sense, as early development targets because they do not place the whole slide image in the role of primary diagnostic medium, tend to involve lower slide volumes and less time stringency. The slides are made in histology, sent to pathologist for diagnosis and are then sent to the "slide room' for management after sign out, and the WSI operation is placed in (or associated with) the slide room. In our experience, basic infrastructure that makes the applications work well include bar coding with readers in histology, slide room and on the imagers, a standard imaging request mechanism and skilled imaging technicians.

Our main interest however, has been in the pre-diagnostic area. Well-known applications in this space include "virtual" distribution of control slides and immunohistochemistry slides [6]. However, the most important pre-diagnostic situation would be placing WSI robots in the clinical histology lab and incorporating specific imaging protocols (and image analysis) in specific histology protocols for specific specimen types. This would greatly increase the volume (and value) of WSI and would drive further investment and investigation. This activity is at robust pilot phase at MGH.

## Results

When one places a WSI robot at the end of the slide creation process in clinical lab (after the cover slipper and before slides are sent to the pathologist), one puts a number of stresses on the system. In particular, the system must be reliable (at least as reliable as the rest of the histology lab), and it must be fast (at least fast enough to keep up with the throughput of the lab). This merges the performance of the imager to the performance of the lab. For example, if the lab organized to generate a bolus of 500 slides at nine AM, no device, no matter how fast, will be fast enough. On the other hand, if a lab is organized to generate a continuous flow (or mini-batches) of 15 slides every 15 minutes. Modern, high end imagers can "keep up" with this flow with minimal delay of slide delivery to the pathologist if a subset of cases is presented to the imager based on specimen type and processing rules *and if the slides are well made *(see below).

Placing a WSI robot in a clinical histology lab quickly demonstrates a necessary symbiosis between histology and imaging. The *histology lab plus the WSI robot need to be considered as single imaging device*, or, to put this another way, the WSI robot needs to be considered as just another automated histology device (like a processor, stainer or cover-slipper). WSI robots need to be reliable and fast (a large number of slow devices might work, but most histology labs have limited space), but laboratories will likely need to change to automated, mini-batch processes orchestrated by the LIS use bar coding to track and direct slides and incorporating the decision to image into the specimen type and the histology orders.

There is another interaction between imaging and histology that possibly more imaging robots create better images, and create them faster and more reliably, if they are presented with high quality tissue slides. In other words, the quality of the image is a function of the quality of the slide. The histology parameters of tissue placement, the flatness and thinness of sections, their staining, and the quality of cover slipping and label placing all affect image quality, capture speed and reliability. There are good reasons for this, details will be presented by Dr Yukako Yagi at her scientific presentation and paper at this conference. Imaging and Histology are two faces of the same thing – they cannot be meaningfully separated.

## Conclusion

Imaging in pathology does not begin with WSI robot but in the grossing room and histology lab. As more and more imaging devices are placed in histology labs, the relationships and histology parameters such as fixation, cutting and staining, and imaging parameters such as illumination and dynamic range will become increasingly studied and, over time, will allow us not only to take better pictures, but to actually see more detail and structure.
